# Online public response to emergency department diagnostic error report: A qualitative study

**DOI:** 10.1111/acem.15047

**Published:** 2024-11-12

**Authors:** Timothy J. Sanford, Pranav Kaul, Danielle M. McCarthy

**Affiliations:** ^1^ Department of Emergency Medicine, Feinberg School of Medicine Northwestern University Chicago Illinois USA

**Keywords:** diagnostic error, emergency care, misdiagnoses, public opinion

## Abstract

**Background:**

The 2022 study on diagnostic error in the emergency department (ED) published by the Agency for Healthcare Research and Quality (AHRQ) reported that one in every 18 ED patients is misdiagnosed. The report was methodologically critiqued by emergency physicians and researchers. However, little is known about public perception of error in the ED. We sought to characterize public response to the publication.

**Methods:**

A search was conducted for online news articles published December 2022 reporting the diagnostic error study and containing “public comment” sections. Verbatim comments and relevant characteristics were collected. Three coders completed content analysis and resolved any differences. Descriptive statistics and themes are reported.

**Results:**

Fifteen online articles were reviewed; three had public comment sections (*New York Times, DailyMail,* and *Boston Globe*). There were 553 unique user comments; 293 were original comments (53%) and 260 were replies to comments (47%). The 260 replies were in response to 113 original comments, with the remaining original comments having 0 replies (*n* = 180). Of the 202 commenters who identified a personal role in a health care encounter, 70 (35%) identified as patients and 68 (34%) identified as physicians. Comments centered on seven major themes: (1) negative personal experiences, (2) reframing study conclusions, (3) sense of decline in training standards, (4) internal stressors impeding ED diagnostic accuracy, (5) external stressors impeding ED diagnostic accuracy, (6) suggested solutions, and (7) role of the ED in diagnosis.

**Conclusions:**

The news coverage of the diagnostic error study provided individuals a platform to share their perspectives. Many comments reflected a nuanced understanding of the role of emergency care and the stressors of the ED environment. Despite questions about the report's accuracy, there were many individuals who shared personal negative experiences suggesting that the public may feel directly impacted by error in the ED.

## INTRODUCTION

Diagnostic errors are ubiquitous in medicine, found across every medical specialty and encountered at least once by nearly all American adults that interact with the health care system.^1^ Incorrect diagnoses, missed diagnoses, and delays in diagnosis contribute to negative health outcomes, making diagnostic error a significant barrier to optimized patient health.^2^ Although medical error, and diagnostic error specifically, is increasingly studied as a cause of adverse health outcomes, there remains a significant gap in our knowledge of individual patient opinions and experiences of diagnostic error.^3^, ^4^, ^5^

While diagnosis is a fundamental process in medicine, the nature of the emergency department's (ED) relationship with diagnosis is quite unique. The practice of emergency medicine “is not ‘diagnosis’ oriented” and the primary goal is more often “real‐time identification and treatment of life‐threatening conditions.”^6^ These factors make the study of diagnostic error in emergency care particularly challenging. In 2022, the Agency for Healthcare Research and Quality (AHRQ) published a report, “Diagnostic Errors in the Emergency Department: A Systematic Review,” by Newman‐Toker et al seeking to quantify and characterize diagnostic errors in the ED setting. Ten leading EM professional organizations published a letter expressing concerns regarding the references cited by the Newman‐Toker report, data interpretation, and the accuracy of the study's conclusions.^7^ Following publication the report received widespread coverage in popular news sources for the conclusion that 7.4 million Americans are misdiagnosed in EDs each year, leading to 50 deaths caused by diagnostic error in the average American ED.^8^

Among the concerns of the EM professional organizations was that the publication of the report would negatively impact not only the workforce in emergency medicine but also the perceptions held by those seeking emergency care. Extensive media coverage of the diagnostic error report at the time of its release in 2022 provides a unique opportunity to explore public opinion. Using online comment sections, individuals from varied locations and backgrounds can share their responses to news stories as they emerge. This descriptive qualitative study aims to use online comments to characterize public response to the 2022 diagnostic error publication, providing insight to the general population's view of diagnostic error in the ED.

## METHODS

### Study design

This was a retrospective qualitative analysis of publicly available comments to online news articles published on the topic of the AHRQ published report entitled “Diagnostic Errors in the Emergency Department: A Systematic Review.” This study was approved by the institutional review board as non–human subjects research.

### Data collection

A search was conducted for online news articles published in December 2022 reporting on the Newman‐Toker et al study and containing “public comment” sections. Using two different internet search engines (Google and the newspaper section of Nexus Uni), search criteria were set to include results from December 14, 2022, to December 31, 2022. Key terms included “misdiagnosis,” “diagnostic error,” “AHRQ,” and “emergency room/department.” Our search was limited to news articles rather than commentaries as the objective was to analyze the initial public response to the news reporting of the report's release, rather than analyzing responses to the report's interpretation. The search strategy focused on general media coverage rather than coverage aimed at specific professional audiences (e.g., Bioethics.com, Sokolove Law). For included articles, verbatim comments, commenter characteristics (region, username), and comment characteristics (original or response comment, number of likes) were collected. Comments and usernames were additionally coded for self‐identified role as health care provider (physician, nurse, other), patient, or family/friend of health care provider or patient. When available, the commenter's self‐reported location was also collected.

### Data analysis

Content analysis of individual comments was performed using a constant comparative qualitative approach with three independent coders in an iterative fashion. The coding team consisted of the three authors (DMM, PK, TS) with varying levels of experience in qualitative analysis and exposure to emergency medicine. One of the coders was a board‐certified emergency physician (EP) with extensive experience in qualitative analysis; one was a resident trainee in EM with prior experience in qualitative analysis and the third coder was a medical student with no prior experience in qualitative analysis. Each coder independently reviewed and coded the data followed by group meetings to reconcile codes. Comment text was analyzed using an inductive and dynamic approach to structure coding categories as new content emerged during the analysis. An audit trail and memos were maintained throughout the coding process. Codes were reconciled through discussion then grouped into common themes.

The comment characteristics of the total sample (e.g., original vs. reply comment, commenter location, number of likes) was summarized. Data were analyzed using Stata 13.1 (StataCorp).

## RESULTS

Fifteen articles from major online news sources were identified through online search. Twelve articles were excluded because they did not have public comment sections (CNN, CBS News, FOX LA, USA Today, Kaiser Family Foundation Health News, HealthDay, Katie Couric Media, KNewz, NBC news affiliates [KOMU, KWWL], Hartford Courant, Baltimore Sun, MailOnline). Three articles with public comment sections were included in the final sample: (*New York Times* [NYT], *DailyMail* [DM], and *Boston Globe* [BG]). NYT's “ER Doctors Misdiagnose Patients with Unusual Symptoms” was published December 15, 2022, and received 426 user comments,^9^ while DM's “Quarter of a million Americans die every year after being misdiagnosed in ER, federal study suggests” published December 16, 2022, received 119 comments.^10^ BG's “ER Doctors Misdiagnose Patients with Unusual Symptoms” was identical to the NYT article and credited its author.^11^ The BG article was published December 15, 2022, and received nine comments, notably without displaying information regarding “likes” or commenter location.

### Comment/commenter characteristics

A total of 554 total user comments were collected from the NYT, DM, and BG articles; one duplicate comment was excluded making a total of 553 unique comments included for analysis. Of the 553 comments, 293 (53%) were original comments and 260 (47%) were comments made in response to the comment of another individual (or “replies”). The 260 replies were in response to 113 original comments, with the remaining original comments having no replies (*n* = 180). Comments showed significant variation in length from single‐word responses to full paragraphs and varied in the number of likes (or “recommends”) from zero to 554 with a median value of 11. Comments included a wide range of subject matter explored further in the thematic results below.

Commenters contributed from all major geographic regions of the United States, while a minority of commenters were from outside the United States or listed a custom placename (Table 1). A total of 202 comments (37%) were contributed by writers who identified one or more specific roles that they have participated in during interactions with the health care system. A plurality of comments were from commenters who identified themselves as patients (*n* = 70, 35% of those with role) and 69 (34%) from commenters who identified their role as a physician. (Table 1).

**TABLE 1:** Number of individuals from each region of origin derived from self‐reported geographical locations for NYT and DM commenters (left) and number of individuals in NYT, DM, and BG comments who self‐reported each of the identified roles in health care system interactions (right).

Regions of origin of comment		Self‐reported roles	
Region of origin	*n* (%)	Healthcare role	*n* (%)
Northern United States	135 (25)	Patient	70 (35)
Southern United States	78 (14)	Friend/family of patient	31 (15)
Midwestern United States	66 (12)	Physician	69 (34)
Western United States	131 (24)	Nurse	12 (6)
“USA”	42 (8)	Other medical professional	9 (4)
International	30 (6)	Friend/family of medical provider	4 (2)
Fake/custom (e.g., “a logical world,” “earth”)	58 (11)	Multiple roles	7 (3)
None provided	4 (<1)		

Abbreviations: NYT, *New York Times;* DM, *DailyMail;* BG, *Boston Globe*.

### Themes

Twenty‐five unique codes were derived and defined in the codebook during the iterative review process. These 25 codes were then grouped into seven overarching themes present throughout the 553 total user comments. The themes (and their frequencies) were: (1) negative personal experiences (20%), (2) reframing study conclusions (12%), (3) sense of decline in training standards (16%), (4) internal stressors impeding diagnostic accuracy in the ED (20%), (5) external stressors impeding diagnostic accuracy in the ED (21%), (6) suggested solutions (20%), and (7) role of the ED in diagnosis (5%). Some comments related to multiple themes. Themes are detailed with representative comments below.

#### Negative personal experiences

From the 553 comments analyzed, there were 111 with stories sharing negative personal experiences in ED settings (70 by patients/families, 10 by physicians and health care providers, and 31 by writers without role). Commenters relayed specific instances of misdiagnosis in the ED and their impact on patients. For example, as one commenter wrote:

My 30 year old daughter just died from misdiagnosed long QT syndrome. A 95% success rate does not feel adequate if you are the mother of someone in the 5% misdiagnosed. She was female, was vomiting a lot, and had a previous seizure. A CT scan had been done, but no one thought to do an EKG.

In addition to misdiagnoses, others commented on cases of diagnostic delay:

I Know [sic] personally of 3 instances of misdiagnosis by ER doctors. 2 were appendicitis that became life‐threatening after both were sent home and eventually involved lengthy ICU and hospital stays. The third was a case of gall bladder stones that were overlooked as a reason for pain and caused the person lots of $$ in obtaining the correct diagnosis and treatment.

Several commenters similarly questioned if an adequate breadth of diagnostic possibilities were explored in their care and attributed the errors in part to doctors that “… DO NOT LISTEN THOROUGHLY TO WHAT PATIENTS ARE TELLING THEM.” Patients and families who shared interactions with physicians overwhelmingly demonstrated concern that EPs are not valuing their input. One patient's “caregiver” directly connected this feeling to experienced diagnostic error:

The pressure in ERs is at full boil. But even so, in the most quiet of ERs, I've seen diagnoses blown by practitioners who refuse to hear what patients and families are telling them.

While the majority of personal anecdotes (*n* = 172) shared by the general public in the NYT, DM, and BG comment sections were negative experiences relevant to ED care and diagnostics (*n* = 111, 65%), a minority shared positive experiences (*n* = 23) in the ED (or positive/negative diagnostic experiences in other health care settings (*n* = 38)).

#### Reframing study conclusions

Initial reactions to the reported error rates and millions of annual misdiagnoses varied significantly among commenters. Sixty‐seven total comments approached the reports with reinterpretations of the diagnostic error study findings (five by patients/families, 22 by physicians and health care providers, two by provider family, 38 writers without role). Among the reinterpretations were those that discussed the misdiagnosis rate as rather reassuring. Many comments noted the unique challenges facing ED providers as important context when reacting to the report. One such commenter found the rate of diagnostic accuracy to be the key take‐away, suggesting a change to the article's title:

New headline for this article: “ER doctors correctly diagnose more than 95% of patients even under horrific working conditions.”

Others were seemingly unsurprised by the reported error rate, as one person noted, “Doctors are not Gods,” while another commenter similarly stated, “Everyone, including board certified doctors, make mistakes.” Other commenters reframed the reported conclusions through criticism of the AHRQ study's approach, methods, or applicability to American EDs. For example, one stated: “As a layperson, the study seems easily flawed by the number variables that could impact the results.” Several others commented specifically on the data sources used by the Newman‐Toker et al study, for example:

How can the authors equate findings of Emergency Services in Europe, where some countries do not even have residency training in Emergency Medicine. These findings are skewed and smell of bad publicity against the medical profession.

Together, comments highlighting the rate of accurate diagnosis, error as a natural occurrence, and questioning study methods challenge the Newman‐Toker study's conclusions as reported in NYT, DM, and BG. Nineteen total comments included evaluation of the study methods rather than just the reported conclusion.

#### Declining training standards

Eighty‐nine comments detailed concerns regarding the current training standards of medical professionals (12 by patients/families, 20 by physicians and health care providers, three by provider family, 54 by writers without role). One frequently mentioned concern was a perceived change in medical school admissions standards, the quality of physician education, and the level of training required of ED staff. While one commentor implicated an “elimination of merit‐based medical degrees,” another simply asked, “What do you expect when their [sic] are no standards anymore for admission to medical school[?]” Others focused more on the training received during physician education than the admissions process. For example, one self‐identified physician expressed concern over the training received by EPs in particular:

More hands‐on training is required. Physicians in training (interns & residents) need to go back to 27 hour shifts & taking care of a patient from presentation to stabilization … Today's residents can cite literature but they cannot apply it appropriately. Too many ER physicians are being trained in second rate programs just to produce them.

Several other commenters identified training as a possible factor uniquely connected to the ED. One individual cited their experience with ED providers at academic centers:

The problem is that in teaching hospitals, the ER is staffed by residents and overseen by a fellow. Nary a fully‐trained physician in sight.

In addition to comments on medical school and residency training of physicians, there were numerous comments about the roles of nonphysician medical staff in the ED. One commenter connected hospital finances to nonphysician roles, proposing that “physicians being replaced by Advanced Practice Providers because they are cheaper may be another factor” impacting diagnostic error rates.

#### Internal stressors impeding diagnostic accuracy in the ED

ED‐specific stressors were also highlighted as possible contributors to diagnostic error in 113 total comments (18 by patients/families, 32 by physicians and health care providers, 63 by writers without role). As one person wrote, “An ER can be a pressure cooker. So much is happening at any moment in time,” another similarly adds, “The ER right now is not a great place to be, overcrowded, understaffed, rushed, etc. It's begging for mistakes.” The use of EDs as primary care locations was frequently mentioned as a contributor to diagnostic error, as was crowding due to the increased use of the ED for psychiatric presentations. For example, as one individual explains their perspective:

Over the past several decades the ERs have become many people's source of primary care, a dumping ground for mental health patients and a repository for patients who need care in rother [sic] facilities and unfortunately where there may or may not be a bed with this particularly true for psychiatric patients, child and adult.

Other ED‐specific factors raised often in the comments include time‐related pressure and a paucity of resources and relationship wherein EPs have “snapshot in time view of the patient.” Others highlighted that these stressors are not limited to the EPs, but also to patients who are “presenting at the ER are at the worst of their life, many times unconscious. How do you expect them to be articulate?” In summary, frequently mentioned aspects of the ED included overcrowding, a lack of time or resources, communication difficulties, and complex diagnoses.

#### External stressors impeding diagnostic accuracy in the ED

While many comments described factors within the ED that may affect diagnostic accuracy, 114 other comments faulted factors outside of the ED environment (15 by patients/families, 24 by physicians and health care providers, and 75 from writers without role). Of these extrinsic stressors, corporate and for‐profit financial structures in health care were mentioned most frequently as contributing to diagnostic error through their impact on ED care. For example, one commenter wrote, “I blame hospital suits and their cost cutting nonsense for errors,” while another cited a “productivity‐driven, rapid‐fire system.” As one commenter related these issues to staffing writing, “Understaffed hospitals because of budget constraints while the so‐ called hospital ‘executives’ make $13 million,” others noted the possible role of private equity and corporations:

ER physician practices are being acquired by private equity at a rapid clip. Having a heavily levered finance bro calling the shots in regards to staffing and compensation is a sure recipe for creating an environment where patients are likely to be misdiagnosed.

Related to the comments about the financial structure and corporate operation of health care, several commenters pointed out the cost of testing as a barrier to diagnostic accuracy. As one self‐identified EP wrote:

Studies like this don't and cannot account for the cost of over‐diagnosing, but end up increasing pressure on docs to run even more testing that an overburdened health system cannot handle.

Similarly, another physician asked, “Should I be ordering MRIs on all patients with no red flags and normal vitals who can walk? (Who's paying for that?)” In addition to finance‐related factors discussed above, other extrinsic stressors often discussed included political influences, gun violence as a cause of increased stress on EDs, and the COVID‐19 pandemic as a factor that impacted diagnosis.

#### Suggested solutions

While many comments explored causes for diagnostic error in the ED, 108 total comments focused on possible solutions (20 by patients/families, 20 by physicians and health care providers, one by provider family, 67 by writers without role). Suggestions offered by commenters included changes at the patient level and provider level and for the health care system as a whole. Patient‐level solutions ranged from tips for self‐advocacy and interacting with ED providers to suggestions of avoiding the ED altogether (e.g., “Do not go to the ER unless you are dying.”). A large number of patient‐directed solutions involved information sharing, as one commenter suggested, “Tell the staff ALL of your symptoms and answer questions honestly. Do NOT be offended by questions! Be your own advocate.”

Many other comments provided suggestions for ED physicians to improve diagnostic accuracy. Solutions offered for ED physicians overwhelmingly pertained to communication skills with patients, as one self‐identified patient wrote, “It would help if doctors listened to the patient in the ER,” while other commenters suggested more attention to patients as one comment shared that a “big improvement would occur if providers would stop looking at the screen, ask the patient and caregivers questions, and really listen to the answers.” In addition to communication improvements, other suggestions for providers focused on resources and processes used for making diagnoses, such as improved incorporation of technology in diagnostics. For example, one individual stated that ED physicians “should keep their laptops handy,” while another similarly added, “a Google search of those two symptoms would have came up with the correct diagnosis.”

System‐level solutions often proposed an increased role for innovative technologies in diagnostics. One commenter suggests “an MRI machine in every emergency room.” Others mentioned artificial intelligence as a mechanism to improve diagnostic accuracy, as one commenter suggests “AI should definitely be used to help doctors and get to a diagnoses [sic] in a reasonable amount of time.”

Other sweeping health care policy and redesign changes were recommended including universal health care, increased access to primary care, and other financial and health care access initiatives. For example, one individual contributed:

Giving people access to affordable health care thus allowing doctors more time and resources for actual emergencies would be a good place to start.

Notably, system‐level solutions (66) appeared most frequently in the comments section compared to physician‐level (20) and patient‐level (22) solutions.

#### Role of the ED in diagnosis

Importantly, discussion developed regarding the role of emergency medicine within the health care system, specifically questioning if diagnostic accuracy is a relevant measure to determine an ED's success. This theme was less prominent than others, with 26 total comments (three by patients/families, 10 by physicians and other providers, 13 by writers without role). User comments provided insight to individual perspectives of the ED's unique relationship with diagnosis compared to the rest of the health care system. For example, one self‐described EP offered their perspective:

If someone is discharged from the ER with a benign diagnosis, and then returns, without any harm, and gets the right diagnosis and treatment, that's ok. It may be inconvenient, but it's not harmful.

Others contributed similar points to the discussion, as one individual noted, “most people don't [sic] realize the ER is to stabilize you” while another added their interpretation of the ED's role: “It is to RULE OUT Life or limb‐threatening diagnoses … Then, it falls on the rest of the medical system.” As mentioned previously, the conversation surrounding the ED's relationship with diagnosis involved fewer comments than other major themes, but the subject matter was unique. Most comments in this category noted that the report's conclusions were likely due to a larger issue in health care with the “blame” falling on the ED or discussed whether diagnostic accuracy is an appropriate goal for ED care.

## DISCUSSION

We analyzed user comments from public comment sections of three popular online news sources reporting the Newman‐Toker et al 2022 report on diagnostic error in EDs to understand public perception of ED diagnostic error as well as the general public's reaction to the published report. Online comments on news media sources have previously proven useful^3^, ^12^, ^13^, ^14^ to assess public opinion of topics in medicine and we similarly found that this analysis offered useful information from multiple perspectives about public awareness and perceptions of diagnostic error.

Overall, the comments reflected a sense of alarm about the frequency of diagnostic errors cited by the report; however, most individuals did not express surprise. Public comments frequently demonstrated a tone of dissatisfaction with the reported error rates, possibly related to existing perceptions of the American health care system prior to the report. This is consistent with Gallup survey data updated just before the report's publication in December 2022, in which the majority of participants gave noncomplimentary ratings to U.S. health care for the first time since they began collecting this data in 2001.^15^ However, a portion of public response to the report's conclusions took a more positive approach, by emphasizing the rate of correct diagnoses, by challenging the study itself, or by questioning the applicability of diagnostic metrics in the unique ED setting as seen within the reframing study conclusions theme. It is important to note that the commenters posing more nuanced questions about the value of the metrics or positive perceptions were oftentimes those who worked within the health care system. Differing views from those questioning versus affirming the report findings may reflect differing priorities of care and definitions of health care quality between physicians and patients. For example, previous work has found that top priorities for patients often do not appear on provider priority lists^16^ while investigations into factors associated with “quality care” have found that physicians place greater emphasis on factors such as motivation, knowledge, and skill whereas patients are more likely to associate good interpersonal skills with quality care.^17^ The comment sections therefore provide a potential space in which physicians can discuss the reported findings with members of the general public and potentially bridge a gap in the evaluation of ED care between patients and providers.

Among the most common comments were personal patient experiences, conveyed to relate to or to counter reported rates of misdiagnosis. Most of the experiences shared were negative in tone. Categorized under the negative personal experiences theme, these comments included misdiagnosis and adverse patient–provider interactions in the ED, showing that the frequency of error in the ED is felt to directly impact many individuals. Although the stories shared were disproportionately negative, this proportion hopefully represents a sampling bias overestimating the true rate of article readers who personally experienced diagnostic errors in the ED. However, the presence of the negative comments in the public domain accompanying the article may further influence the impression of the ED for readers who peruse the comments section.^18^, ^19^ Prior work exploring patients’ reactions to a 2014 study on diagnostic error in the outpatient setting had similar results with 27% of their sample sharing negative personal stories.^3^

In the above‐referenced study, Giardina and colleagues^3^ suggested the awareness of existing rates of diagnostic error may indicate that the general public is invested in improving the care they receive through “willingness to participate in patient safety and engage in diagnostic error improvement activities.” We share that assessment as commenters on the articles studied seem eager to engage in dialogue about the factors contributing to error and to suggest potential solutions. The causes explored were wide ranging, but perhaps most notably, many comments in the external stressors impeding diagnosis theme reflected a nuanced understanding that an individual ED does not work in isolation but rather is part of a larger hospital system working within the U.S. health care system. In their response to the diagnostic error report, EPs Drs. Kelen and Kaji emphasize that diagnosis should not be the priority in emergency settings and that overcrowding in EDs is likely the most significant factor adversely impacting patient safety.^6^ We found that those in the public comment section share a similar response, as overcrowding was frequently mentioned within the theme of internal stressors impeding diagnosis as a contributor to diagnostic error or as a cause of negative personal experiences. A portion of commenters seen within our theme of role of the ED in diagnosis also questioned whether perfecting diagnosis is an appropriate goal in emergency medicine. Although some of these comments were from physicians, they were also made by patients, and these similarities between ED physician commentary in an academic medical journal and opinions expressed in public commentaries further emphasize commenters’ understanding of and willingness to engage in obstacles facing ED care.

A recent interview‐based study by Mangus et al.^20^ revealed priority categories of problems and necessary change according to physicians, nurses, and patients. Included among the major “vulnerability” categories discovered were issues with communication, obtaining useful histories, and ED system and organization. Our work confirms these prior findings with communication being frequently mentioned within the negative personal experiences theme and the suggested solutions theme wherein patients often mention rushed interactions with physicians and suggest better listening and clearer explanations of their care. The comments within our theme of internal stressors impede diagnostic accuracy in the ED correlate with the Mangus‐derived categories as well, including mentions of staffing and patient factors affecting communication and history‐taking. Interestingly, user comments in suggested solutions referenced health care system–level changes more frequently than other levels of change to address ED diagnostic error. One commonly expressed opinion was that switching to a national universal health care system may decrease medical error and improve patient experiences. Commenters mentioned improved cost, ease of information sharing, and the successful implementation of universal health care systems in other nations as motivations for this suggestion. These comments may reflect a recent shift in public opinion as Gallup studies revealed 57% of Americans view health care coverage as the government's responsibility, whereas only 42% did 9 years prior in 2013.^21^ More recent data from Gallup show a continuation of this trend since 2022.^15^ In addition to universal health care, commenters frequently suggested an increased role for AI in diagnostics, likely influenced by the highly publicized development and launch of ChatGPT by OpenAI in November 2022 just prior to the report's publication in December. However, there was often tension or disagreement between what different individuals valued as solutions. For example, the many suggestions for AI and “keep the laptop handy” were in tension with those requesting that providers “stop looking at the screen” and “listened to the patient.” This tension further underscores the challenge of developing patient‐centered solutions to the issue.

## LIMITATIONS

While this study includes a large number (553) of public comments from individuals of diverse professional and geographic backgrounds as self‐indicated, a primary limitation to its applicability to public opinion is the inclusion of articles from only three news sources. The diagnostic error report received wide news coverage as demonstrated by the list of articles that did not have comments sections including CNN, FOX, and USA Today, which, in combination with the NYT and DM, are all in the top fifteen most used online news sources in the United States according to data from SimilarWeb.^22^ Therefore, it is difficult to know how the public receiving this news from sources outside of the sample, beyond the two week search time frame, or choosing not to comment, perceived the report. While public comment sections in online news media sources are a platform for readers to share their opinions, many news sites no longer allow public comments.^23^, ^24^, ^25^, ^26^ Additionally, the NYT is behind a “paywall” requiring a subscription to access most articles. Despite this limitation, NYT reaches a wide audience, with 2012 readership data showing 32% of individuals aged 18–29, 31% aged 30–49, 21% 50–64, and 12% over 64. The same data show that NYT readers are represented across income levels and political ideologies.^27^ While together the NYT, DM, and BG articles reach a diverse set of readers, online comments still limit our assessment of public opinion to those who read news articles online and may not be generalizable to other populations. The comments only include limited, self‐reported demographics (e.g., region of commenter), limiting analysis and a large proportion of commenters were medical professionals. There are numerous other platforms on which the public can now comment including social media outlets (e.g., X). Thus, limitation to more traditional online news outlets may reflect only a subgroup of the commenters engaging on the topic in public forums more generally and some of these comments may have had other motivations than contributing to productive dialogue, such as “trolling.” Lastly, we did not analyze any commentaries or opinion articles published following the original news articles reporting the study. Inclusion of online comments in response to media commentaries with titles such as “ER doctors are not your enemies” (NBC)^28^ and “A Study Sounds False Alarm About America's Emergency Rooms” (WSJ)^29^ may have offered a different perspective. However, they were excluded due to concern about including comments on commentaries rather than news reporting and the potential for greater focus on the scientific methodology and previous coverage rather than on the diagnostic error report findings.

## CONCLUSIONS

Commenters contributing to the *New York Times*, *DailyMail*, and *Boston Globe* dialogue on diagnostic error in the ED demonstrated a nuanced understanding of potential causes of error, citing stressors within the ED such as crowding and staffing issues as well as external factors including federal policies and a corporatization of health care. Many comments identified perceived obstacles to error‐free care in the ED, offered solutions to reduce error, or detailed negative personal experiences related to ED care. The nature of many comments present in all three of the *New York Times*, *DailyMail*, and *Boston Globe* reinforce the perception that ED care is prone to diagnostic error or negative outcomes. Previous work on the influence of user comments on readers indicate that the tone of user comments significantly impacts a reader's take‐aways from an article.^15^, ^16^ In the setting of widespread media coverage for the Newman‐Toker et al diagnostic error report, publications such as those by *New York Times*, *DailyMail*, and *Boston Globe* may have contributed to unfavorable perceptions of the ED. As such, given the media's known influence on public opinion, additional research should be conducted on the general public's perception of ED care and whether this perception influences decisions to seek or postpone treatment.

## CONFLICT OF INTEREST STATEMENT

DMM receives funding from National Institute on Aging, National Institute on Drug Abuse, National Center for Complementary and Integrative Health, and the Agency for Healthcare Research and Quality. TJS and PK declare no conflicts of interest.

## Data Availability

The data that support the findings of this study are available from the corresponding author upon reasonable request.
